# "ATR activation in response to ionizing radiation: still ATM territory"

**DOI:** 10.1186/1747-1028-1-7

**Published:** 2006-05-17

**Authors:** Myriam Cuadrado, Barbara Martinez-Pastor, Oscar Fernandez-Capetillo

**Affiliations:** 1 Genomic Instability Group, Spanish National Cancer Center, Madrid, Spain

## Abstract

Unrepaired DNA double-strand breaks (DSBs) are a major cause for genomic instability. Therefore, upon detection of a DSB a rapid response must be assembled to coordinate the proper repair/signaling of the lesion or the elimination of cells with unsustainable amounts of DNA damage. Three members of the PIKK family of protein kinases -ATM, ATR and DNA-PKcs- take the lead and initiate the signaling cascade emanating from DSB sites. Whereas DNA-PKcs activity seems to be restricted to the phosphorylation of targets involved in DNA repair, ATM and ATR phosphorylate a broad spectrum of cell cycle regulators and DNA repair proteins. In the canonical model, ATM and ATR are activated by two different types of lesions and signal through two independent and alternate pathways. Specifically, ATR is activated by various forms of DNA damage, including DSBs, arising at stalled replication forks ("replication stress"), and ATM is responsible for the signaling of DSBs that are not associated with the replication machinery throughout the cell cycle. Recent evidence suggests that this model might be oversimplified and that coordinated crosstalk between ATM and ATR activation routes goes on at the core of the DNA damage response.

## Introduction

Accumulation of DNA damage at the cellular level is linked to a number of phenotypes at the organism level including progeria, neurodegeneration and cancer. Therefore, understanding how cells respond to lesions in their DNA has become a central area of research in biomedical sciences. In particular, significant effort has been made in elucidating the components and mechanisms of the signaling pathways that alert about the existence of DSBs [1]. Our current understanding is based on a model with two alternative routes. If DSBs arise at the replication fork (the still not fully defined concept of "replication stress"), activation of the ATR pathway is responsible for the signaling of damaged DNA. Alternatively, and in agreement with its general role in cell-cycle checkpoints [2], ATM is responsible for the signaling of other types of DSBs that are not restricted to replicating cells. In addition to ATM and ATR, which are rapidly recruited to the break sites and are the earliest transducers of the DNA damage response, the strength of each pathway is reinforced by the subsequent activation of an additional downstream kinase. Specifically, whereas ATM-dependent responses are amplified by the activation of the kinase Chk2, ATR-dependent activation of Chk1 takes place in response to "replication stress".

The two types of DSBs can artificially be mimicked in the laboratory. Typically, chemicals such as hydroxyurea (HU) or aphidicolin are used to induce activation of ATR, and exposure to ionizing radiation (IR) for activation of the ATM pathway. In addition, the levels of phosphorylation of ATM and ATR targets serve as a quantitative readout of the kinase activities. For instance, Chk2 and Chk1 phosphorylation are regularly used as a measure of ATM and ATR activation status, respectively. However, a number of studies have previously reported that IR, which supposedly only activates ATM, also leads to noticeable phosphorylation of Chk1. One possible interpretation is that this phosphorylation is derived from "accidental" phosphorylation of Chk1 by ATM, particularly at the high doses of IR used in this type of experiment [3]. However, both Chk1 and ATR are known regulators of the G2/M checkpoint in response to IR. [4-6], arguing in favor of a functional role of Chk1 phosphorylation in radiation responses. Moreover, ATR, but not ATM, is known to be the main kinase mediating Chk1 phosphorylation. Data now coming from several groups finally resolves this controversy and provides a novel molecular framework to understand the cellular responses to IR [7-10].

## Discussion

### A novel concept: ATM-dependent ATR activity

The key observation in these works is that although ATM and ATR are both upstream of Chk1 phosphorylation in response to IR, the presence of active ATM in the cell is not sufficient and still requires ATR activity for the modification of Chk1. In other words, the data suggest the existence of active crosstalk among ATM and ATR signaling pathways in response to IR which works through ATM-dependent activation of ATR. But how is ATR activity regulated by ATM? One possibility is that ATR would be directly activated by ATM. In this context, and taken that ATM autophosphorylation is supposed to be necessary for its activation [11], it is tempting to speculate that ATM dependent phosphorylation of ATR could similarly stimulate its activity. However, there is no evidence that supports this hypothesis. In addition, whereas pre-treatment of cells with DNA damage stimulates the activity of purified ATM, there is no difference in the *in vitro *activity of ATR whether is purified from damaged or intact cells [12]. Another possibility is that instead of promoting its kinase activity, ATM regulates the accessibility of ATR to its downstream targets. In fact, recruitment to the sites of damage is probably the most critical level of regulation of PIKK activity [13,14]. The evaluation of ATR recruitment to IR-damaged chromatin revealed that ATR relocalization is indeed dependent on ATM [7-10].

### ATM dependent loading of RPA to DSBs

Within the cell, ATR exists in a complex with ATRIP (ATR interacting protein) and defects in ATRIP are associated with concomitant abrogation of the ATR/Chk1 pathway [15]. Additionally, ATRIP recruitment to DSBs is mediated by its interaction with the ssDNA binding complex RPA (replication protein A) [16]. Therefore, ATM dependent recruitment of ATR could be mediated by the regulation of the pathway at any of these upstream steps (ssDNA generation, RPA or ATRIP modification...). Consistently, localization studies supported the idea of the regulation of the ATR/Chk1 pathway at the level of RPA, since RPA redistribution into IR-induced foci was shown to be dependent on ATM. In addition, both ATR and RPA recruitment to IR-induced DSBs were also dependent on MRN (Mre11/Rad50/Nbs1) complex. However, given that the MRN complex recruits ATM to DSBs [14] these data could be conceptually equivalent to the dependence on ATM. In any case, the work of Jazayeri and colleagues showed evidence arguing that a nuclease deficient mutation of Mre11, which they point out has no effect on ATM activation in their experimental conditions, leads to defective RPA localization to DSBs[9]. In summary, these data suggest that ATM dependent localization of ATR to IR-damaged DNA works at the level of RPA loading, likely through the generation of ssDNA. Further work should be done to elucidate how ssDNA generation and RPA loading in response to IR are controlled by ATM.

### ATM and ATR kinase activities: unanswered questions

In contrast to the crosstalk between ATM and ATR that is activated in response to IR, the analysis of the response to "replication stress" published in these recent reports is still consistent with the canonical model and places ATM/Chk2 and ATR/Chk1 in two alternative and non-interacting pathways. Noteworthy, our data (^8 ^and unpublished) consistently detected an increased loading of ATR onto HU-damaged chromatin in the absence of ATM activity. It is possible that both signaling mechanisms compete for the signaling of DSBs during replication so that the absence of ATM could lead to an increased ATR response or *vice versa*. Another intriguing point is the ATM-dependent phosphorylation of Chk2 in S/G2 phase. In our experiments, ATM dependent phosphorylation of its targets occurs whenever a DSB is present (throughout the cell cycle in response to IR or restricted to S phase in response to "replication stress"). However, there is no data that strongly supports a functional role for Chk2 in the signaling of stalled replication forks. Are ATM/ATR promiscuous enzymes that indiscriminately phosphorylate proteins carrying [S/T]Q motifs once they are activated? Is the specificity given just by the accessibility of the substrates at the sites of DSBs? In the light of these facts, delineating the specific *in vivo *functions of newly identified ATM/ATR-dependent phosphorylation events should be a critical avenue of future research.

### Cell-cycle restricted ATR activity: the CDK connection

Finally, an additional and perhaps more crucial point of these recent publications focused on the study of how the activation of the ATR/Chk1 pathway is restricted to post-replicative cells. Two of these reports [8,9] showed that Chk1 phosphorylation in response to IR is restricted to S and G2 cells. Significantly, the development of a flow-cytometry based approach for the evaluation of Chk1 phosphorylation, demonstrated that ATR activity is not uniform throughout the cell cycle. In contrast, Chk1 phosphorylation starts to be detectable in early S and is maximum by the end of G2. Why this pathway is not active in G1 cells remains uncertain. One possibility is simply that members of the pathway are only expressed in S/G2. Consistently, a recent report claimed that ATR is not expressed in non-replicating lymphocytes [17]. However, this restriction seems to be limited to G0 cells, since all components of the ATR pathway (RPA, ATRIP, ATR and Chk1) are present in G1 (unpublished data). Alternatively, it is possible that some of the members of the pathway need to be "pre-activated" in S-G2 in order to be able to function. The absence of ATR activity in G1 followed by a progressive increase throughout S-G2 supports such a view and is reminiscent of the activity of cyclin-dependent kinases. Importantly, the joint work of the laboratories of Steve Jackson and Jiri Lukas showed experimental evidence for such a model. Specifically, the use of the broad range inhibitor of CDK activity Roscovitin led to a reduction in IR-induced Chk1 phosphorylation [9]. Evaluating the molecular mechanisms that connect CDK activity with ATR "licensing" will certainly become an active area of the DNA-damage studies.

## Conclusion

Elucidation of an active crosstalk between ATM and ATR signaling routes is a live example that there is still room for refining canonical models of molecular biology based on exploiting the cases in which the data does not fit with the existing model (the IR-induced G2/M checkpoint, in this case). On the technical level, we expect that the incorporation of flow cytometry protocols[8] onto DNA-damage signaling events will help in our understanding the molecular choreography of cell cycle checkpoints. Finally, the concept of CDK mediated "licensing" of the ATR pathway, nicely supported by S. Jackson's data comes timely in the context of other related works. There is mounting evidence from both system models like yeast [18] as well as human cells [19], that CDK activity coordinates the assembly of the proper repair complexes in different stages of the cell cycle. In particular, suppression of the homologous recombination (HR) pathway of DNA repair in G1 is of critical importance since HR activity in the absence of the proper sister chromatid could easily lead to the generation of chromosome aberrations and translocations. In the DNA damage response field we are used to evaluating how ATM and ATR activities finally converge into silencing of CDK activities and thus activation of cell-cycle checkpoints. These recent studies maybe just the "tip of the iceberg" in the emerging reverse concept; namely that CDKs control cell-cycle specific DNA damage signaling pathways.

## Abbreviations

DSB: DNA double-strand break; PIKK: phosphatidyl-inositol 3 kinase like protein kinase; ATM: Ataxia Telangiectasia mutated; ATR: ATM and Rad3-related; DNA-PKcs: DNA-dependent protein kinase catalytic subunit, HU: hydroxyurea; IR: ionizing radiation; ssDNA: single stranded DNA; RPA: replication protein A; CDK: cyclin-dependent kinase.

## Competing interests

The author(s) declare that they have no competing interests.

## Authors' contributions

MC and BM contributed to the initial draft of the manuscript. OF wrote the manuscript. All authors read and approved the final manuscript.

**Figure 1:**
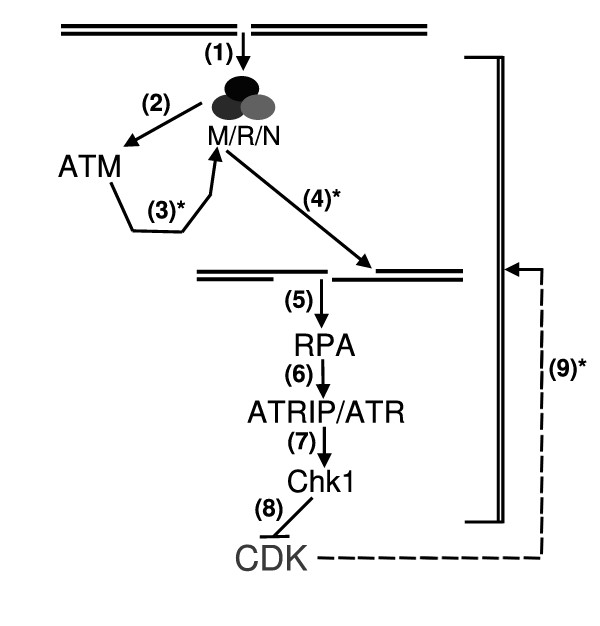
**Sequence of events in ATM→ ATR dependent signaling of DSBs in S/G2 cells**. (1) Recuitment of the MRN complex to the DSB. (2) Recuitment/activation of ATM to DSBs. (3) ATM dependent stimulation of the Mre11-dependent 5' to 3' nuclease activity. (4) Generation of ssDNA. (5) Recruitment of RPA to the generated ssDNA. (6) Recruitment of the ATRIP/ATR complex to ssDNA-bound RPA. (7) ATR dependent activation of Chk1. (8) Inhibition of the specific CDK activity responsible for S and/or G2/M progression; in other words, execution of the checkpoint. (9) Control of the ssDNA/RPA/ATR pathway by CDK activity. The numbers followed by an asterisk (3,4 and 9) point to the less understood concepts of the model: (3)* How does ATM stimulate Mre11 nuclease activity? (4)* To what extent is Mre11 nuclease activity responsible for the ssDNA generation in response to IR-induced DSBs? And, most importantly; (9)* how does CDK activity "preactivate" the ATR/Chk1 pathway?
